# Review on the Applications and Molecular Mechanisms of *Xihuang* Pill in Tumor Treatment

**DOI:** 10.1155/2015/854307

**Published:** 2015-06-10

**Authors:** Qiujun Guo, Jinyin Lin, Rui Liu, Yebo Gao, Shulin He, Xinyao Xu, Baojin Hua, Conghuang Li, Wei Hou, Honggang Zheng, Yanju Bao

**Affiliations:** ^1^Department of Oncology, Guang'anmen Hospital, China Academy of Chinese Medicine Sciences, No. 5 Beixiange, Xicheng District, Beijing 100053, China; ^2^Beijing University of Chinese Medicine, No. 11 North Third Ring Road East, Chaoyang District, Beijing 100029, China; ^3^Beijing Tongren Hospital, Capital Medical University, No. 2, Chongwenmennei Street, Dongcheng District, Beijing 100730, China; ^4^Institute of Medical Information, Chinese Academy of Medical Sciences and Peking Union Medical College, No. 3 Yabao Road, Chaoyang District, Beijing 100020, China

## Abstract

*Xihuang* pill (XH) is a complementary and alternative medicine that has been used in traditional Chinese medicine (TCM) for the treatment of tumors since the 18th century. XH has clinical effects on non-Hodgkin lymphoma, breast cancer, gastric cancer, liver cancer, and bone metastasis. XH can also inhibit the growth of tumor cells and cancer stem cells, prevent tumor invasion and angiogenesis, and regulate the tumor microenvironment. XH is composed of *Ru Xiang* (olibanum), *Mo Yao* (*Commiphora myrrha*), *She Xiang* (*Moschus*), and *Niu Huang* (*Calculus bovis*). Some of the compounds found in these ingredients exert multiple antitumor effects and may synergize with the other ingredients. We aimed to summarize the clinical applications and molecular mechanisms of XH and its chemical composition. This review will provide potential new strategies and alternative perspectives for tumor treatments and basic research into complementary and alternative medicine.

## 1. Introduction

*Xihuang* pill (XH), also called* Xihuang Wan*, is a complementary and alternative medicine used for tumor treatment in traditional Chinese medicine (TCM) since the 18th century. XH was originally developed by Wang Weide and was recorded in the* Wai Ke Quan Sheng Ji* during the Qing dynasty. XH was used for noxious heat with blood stasis syndrome. The prescription contains four TCM ingredients: three blood-activating and stasis-eliminating compounds (*Ru Xiang *[olibanum]*, Mo Yao *[*Commiphora myrrha*], and* She Xiang *[*Moschus*]) and a heat-clearing and detoxifying compound (*Niu Huang *[*Calculus bovis*]). XH was recorded to have effects on treating lung cancer, breast cancer, intestinal cancer, lymphomas, and lymph node metastasis of malignant neoplasms in ancient China.

TCM is very important to tumor treatment strategies in China [1]. TCM is accepted in China to enhance the antitumor effects of conventional therapies, reduce the toxicity of chemotherapy and radiotherapy, alleviate tumor-induced clinical symptoms and cancer pain, and prolong the survival time of postoperational and advanced-stage cancer patients [2]. XH is often used in East and Southeastern Asia countries as an adjunct treatment combined conventional tumor treatment methods such as chemotherapy. XH is usually administered orally in 3 g doses twice a day and sporadically has skin rashes or pruritus as side effects [3].

We aimed to review the antitumor research of XH in both clinical and basic aspects and summarize the antitumor mechanisms of the four components of XH. Prospects and development trends for the application and study of XH are also described. This review may provide new strategies and different viewpoints on tumor treatments and basic research into complementary and alternative medicine.

## 2. XH Has Antitumor Effects When Combined with Conventional Therapies

### 2.1. XH Enhances the Response Rate of Non-Hodgkin Lymphoma

Non-Hodgkin lymphomas (NHL) are a heterogeneous group of lymphoproliferative disorders originating in B-lymphocytes, T-lymphocytes, or natural killer (NK) cells. CHOP (cyclophosphamide, vincristine, doxorubicin, and prednisone) is a first line chemotherapy regimen for NHL, and recent studies indicate that treatment with rituximab and CHOP (R-CHOP) is more effective [4, 5].

When combined with CHOP, XH can enhance the response rate to chemotherapy and prolong survival. Wang et al. [6] randomly and equally divided 60 NHL patients into a treatment (CHOP with XH) or control group (CHOP only). The three-year survival rate in the treatment group was significantly higher than that in the control group (92% versus 78%, resp., *P* < 0.05). Furthermore, patients in the treatment group had a greater clinical improvement in symptoms (e.g., hot flashes or night sweats) and Karnofsky's performance scores (KPS). Since rituximab is not included in the medical insurance drug catalogue in some developing counties such as China, R-CHOP might result in a heavy medical burden to patients with NHL and their families. Therefore, XH could possibly enhance chemotherapy effects, relieve NHL related symptoms, and reduce medical burden.

### 2.2. XH Improves the Efficacy of Conventional Therapy and Regulates Immunity in Breast Cancer

Breast cancer is the most common malignancy in women and the second leading cause of cancer-related death [7]. TEC (taxotere, epirubicin, and cyclophosphamide) and CEF (cyclophosphamide, epirubicin, and fluorouracil) are the most commonly used chemotherapy regimens for breast cancer [8, 9]. Radiation therapies [10], targeted therapies [11, 12], and endocrinotherapies [13] are also recommended in specific situations.

XH has been shown to affect breast neoplasms. Hong et al. [14] explored the application of XH with TEC in patients with breast cancer and found that XH could significantly enhance the two-year survival rate and overall response rate in the treatment group. However, XH did not alter the side effects of TEC. Furthermore, XH regulated T-lymphocyte subsets and improved the immunity of breast cancer patients taking CEF [15]. Breast hyperplasia, especially of the columnar cells, is the earliest histologically identifiable lesion linked to cancer progression [26]. Aside from the tumor, XH can treat benign lesions such as breast hyperplasia and prevent transformation into malignancies [27].

### 2.3. XH Has Clinical Effects on Advanced-Stage Liver Cancer

Primary liver cancer (PLC) is characterised by high mortality rate and poor prognosis [28]. Early stage PLC patients can undergo hepatectomy and their overall survival rate is relatively higher than that of advanced-stage patients [29]. Radiotherapy [30], drug therapy (such as the multikinase inhibitor sorafenib) [31], and interventional treatment (radiofrequency ablation or interventional transcatheter arterial chemotherapy [TAC] with or without embolization) [32, 33] are choices for patients with unresectable or recurrent cancer.

Liu et al. [18] combined XH with TAC in advanced-stage PLC patients. Both the overall one-year survival rate and the short-term response rate in the XH combination group were superior to those in the TAC group. Case reports of stage IV PLC patients also indicate that XH could relive tumor-related symptoms such as cancer pain, fever, or abdominal distention [19, 20].

### 2.4. XH Enhances the Effects of Chemotherapy on Gastric Cancer

Gastric cancer (GC) is one of the leading causes of cancer death worldwide, although geographical variations in incidence exist [34–36]. Treatments for GC include chemotherapy (platinum drugs, fluorouracil drugs, taxanes, and camptothecin) [37], radiotherapy [38], surgery [39], endotherapy [40], and HER-2 targeted therapy [41]. However, the effects of conventional treatment are unsatisfactory, especially in the advanced stages [42].

XH was reported to effectively treat stage IV GC when combined with DCF (docetaxel, cisplatin, and 5-fluorouracil) and significantly enhance the short-term response rate compared with DCF alone (77.5% versus 55.0%, resp., *P* < 0.05) [21]. Although the effect is still limited, XH could be an effective adjuvant GC treatment.

### 2.5. XH Controls Cancer-Induced Bone Pain and Promotes the Effect of Zoledronic Acid on Bone Metastasis

Bone metastasis occurs frequently in patients with advanced-stage cancers, such as lung cancer and breast cancer [43, 44]. The main symptoms of osseous metastasis are cancer-induced bone pain (CIBP) and skeletal-related events (e.g., radiation to bone, spinal cord compression and fracture), which decrease quality of life and an increase mortality [45, 46]. Aside from inhibiting tumor cells, zoledronic acid, together with denosumab, is a possible therapeutic regimen for bone metastasis [47].

TCM was reported to have an effect on bone metastasis and CIBP control [48, 49]. Combined with zoledronic acid or used alone, XH could significantly relive the CIBP in patients with breast cancer. Furthermore, XH could mildly enhance the effect of zoledronic acid on bone metastasis and regulate immunity [16].

## 3. XH Can Relieve the Side Effects of Modern Therapies

Modern therapies provide effective ways to treat tumors but have some toxicities and side effects, such as chemotherapy-induced peripheral neurotoxicity [50], radiation-induced stomatitis [51], or endocrinotherapy-induced menopause-like syndrome [52]. XH could be effective in this field as a natural supplementary medicine. In fact, XH was found to improve the quality of life and Karnofsky's performance score in tumor patients [6] and relieve chemotherapy-induced phlebitis, radiation-induced stomatitis, and endocrinotherapy-induced menopause-like syndrome [17, 24, 25].

XH has been comprehensively used in tumor treatments, both to improve efficacy and reduce side effects of conventional therapies. XH was also reported to have effects on esophageal cancer and brain glioma [22, 23]. The clinical uses of XH are listed in Table 1.

## 4. XH Inhibits Tumor Cells via Multiple Pathways

### 4.1. XH Inhibits the Growth of Tumor Cells

Resisting cell death is the hallmark of cancer [53] and results in tumor growth. Tumor cells use several pathways to suppress apoptosis and acquire resistance to apoptotic agents, such as via expression of antiapoptotic proteins like Bcl-2 [54]. In fact, overexpression of Bcl-2 is a characteristic of drug-resistant tumor cells [55]. Drugs and microRNAs may regulate the Bcl-2 mediated apoptotic resistance [56, 57].

XH could induce H22 cell (mouse liver cancer cell line) and Bel-7402 cell (human liver cancer cell line) apoptosis by downregulating Bcl-2 expression in tumor-bearing mice [58, 59]. XH extract also inhibited the proliferation of human tumor cell lines MDA-MB-231 (breast cancer cell line), SMMC7721 (liver cancer cell line), T24 (bladder cancer cell line), A549 (lung cancer cell line), and LoVo (colorectal cancer cell line)* in vitro* [60, 61]. Therefore, XH inhibited tumor growth both* in vivo* and* in vitro*.

### 4.2. XH Prevents Invasion and Metastasis of Tumor Cells

Tumor cells invade adjacent tissues, which makes it difficult to be completely resected and liable to form metastasis. In the tumor microenvironment, tumor cells downregulate E-cadherin expression and overexpress N-cadherin and vimentin, which weakens intercellular adhesive attractions and facilitates invasion and metastasis. This process is called the epithelial-mesenchymal transition process [62] and could be inhibited by some TCMs [63]. Tumor cells degrade and remodel the extracellular matrix (ECM) by excessively secreting matrix metalloproteinases (MMPs) such as MMP-2 and MMP-9 [64].

XH can inhibit the epithelial-mesenchymal transition and ECM degradation. XH promoted mRNA levels of E-cadherin and suppressed N-cadherin expression in LoVo cells by regulating the ZEB1-SCRIB loop [61]. Additionally, XH had a potent effect on reducing the expressions of MMP-2 and MMP-9 in LoVo cells and 4T1 (mouse breast cancer) tumor-bearing mice [65, 66]. Therefore, XH intervention suppressed the invasion, migration, and metastasis of LoVo cells.

### 4.3. XH Inhibits Angiogenesis

Dysregulated angiogenesis can result in angiogenic diseases and is responsible for solid tumor growth and metastasis. When tumor tissues become hypoxic or hindered by the lack of nutrition, proangiogenic factors, such as vascular endothelial growth factor (VEGF) and nestin, predominate and result in angiogenesis and tumor progression [67, 68]. Bevacizumab or other angiogenesis-targeting drugs could improve the outcome in patients with metastatic cancer [69, 70]. The methanol extract of XH had an antiangiogenic effect on the zebrafish embryo [71] and XH could prevent the expression of VEGF* in vivo* [66].

### 4.4. XH Prevents the Proliferation of Cancer Stem Cells (CSCs)

Since their initial discovery, CSCs have become a formidable challenge to cancer eradication [72]. CSCs can self-renew, give rise to cells that are different from them, and use common signaling pathways. CSCs may be responsible for the resistance of chemotherapeutic agents used to treat malignant tumors and may be the source of cells that give rise to distant metastases [73]. Plant-derived bioactive compounds can play a role in the regulation of CSC self-renewal [74]. XH could regulate and inhibit the growth of LAC (human lung cancer cell line) CSCs* in vivo* and* in vitro* by regulating the Wnt pathway [75].

### 4.5. XH Regulates the Tumor Immune Microenvironment (TIM)

The TIM is complex and composed of immune cells that penetrate the tumor site via blood vessels and lymphoid capillaries [76]. TIM has an immunosuppressive role that involves synergistic suppressive cells, including regulatory T cells, tumor-associated macrophages, dendritic cells, and myeloid-derived suppressor cells (MDSCs) [77]. TIM also expresses immunosuppressive factors (IL-10, TGF-*β*), VEGF, and MMPs to prevent tumors detected from the antitumor immune cells and promote the invasion and metastasis of tumors [78].

Cancer immunotherapies targeting TIM have been developed and used in clinic [79], and TCM has effects on TIM by improving antitumor immunity and reversing immunosuppression [77]. One study found that XH ameliorated immunosuppression and inhibited tumor growth in tumor-bearing mice by reducing the expression of MDSCs [66]. Moreover, the chloroform, ethanol, and volatile oil extracts of XH could enhance the expression of immune system promoting factors (IL-2 and IFN-*γ*) and CD80 on antigen-presenting cells, decrease inhibiting factors (IL-10), and regulate the ratio of T-lymphocytes in a Walker256 (rat breast cancer cell line) tumor-bearing rat model [80–82].

## 5. Antitumor Effects and Pharmacological Studies of Phytochemicals in XH

### 5.1. Olibanum

Olibanum, commonly called frankincense, is the resin exuded from* Boswellia carteri *Birdw. and is used as an incense in religious and cultural ceremonies. Its medicinal properties are also widely recognized, mainly in the treatment of inflammatory conditions, some cancerous diseases, wound healing, and for its antimicrobial activity [83].

Olibanum contains triterpenoids, beta-boswellic acid, and its structurally related derivatives, which might be the most active compounds. Research showed that *β*-boswellic acid, 3-O-acetyl-*β*-boswellic acid, 11-keto-*β*-boswellic acid, and 3-O-acetyl-11-keto-*β*-boswellic acid inhibited DNA synthesis in HL-60 cells (human leukemia cells) [84]. Another study found that tirucallic acid, isolated from olibanum, is an effective Akt inhibitor and resulted in cytotoxic effects on human prostate cancer cells* in vivo* and* in vitro *[85]. *β*-boswellic acid could also inhibit NF-*κ*B signaling, which is identified as an oncogenic factor [86].

### 5.2. Myrrh

Myrrh is the resin from* Commiphora myrrha *Engl. and has been used for centuries to treat internal tumors, obesity, liver disorders, malignant sores and ulcers, urinary complaints, intestinal worms, leucoderma, sinus problems, edema, and sudden paralytic seizures [87]. *β*-caryophyllene is an active component in the essential oils extracted from myrrh and was found to potently induce apoptosis of BS-24-1 cells (mouse lymphoma cell line) accompanied by the activation of caspase-3 in tumor cells [88].

Guggulsterone (GUG) was identified as another major active component of myrrh that has potent inhibitory effects on tumor cells and anti-inflammatory effects by targeting the farnesoid X receptor [89]. Sarfaraz et al. [90] showed that GUG possesses anti-skin tumor effects in SENCAR mice by modulating the MAPK and NF-*κ*B pathways. Xiao and Singh [91] found that* Z*-guggulsterone (an isomer of GUG) inhibited angiogenesis by suppressing the VEGF-VEGFR2-Akt signaling axis. Furthermore, studies indicated that coadministration of GUG resulted in a significant increase in chemosensitivity of multidrug-resistant human breast cancer MCF-7/DOX cells to doxorubicin (DOX)* in vivo* and* in vitro* via Bcl-2 and P-glycoprotein expression inhibition [92].

### 5.3. *Moschus*

*Moschus*, an herbal material used in TCM, was found to induce cell cycle arrest in human cervical carcinoma HeLa cells when combined with* Toona sinensis *[93]. Muscone is one of the active compounds of* Moschus* and has actions on the neural system [94]. Though muscone has not been reported to have any effects on tumors, there are potential mechanisms for tumor therapy. Muscone could significantly enhance cell membrane fluidity and improve the effect of geniposide transport across the human nasal epithelial cell monolayer [95]. Therefore, we hypothesize that facilitating the metabolism and absorption of antitumor drugs might be a mechanism of muscone.

### 5.4. *Calculus bovis*

**Figure 1:**
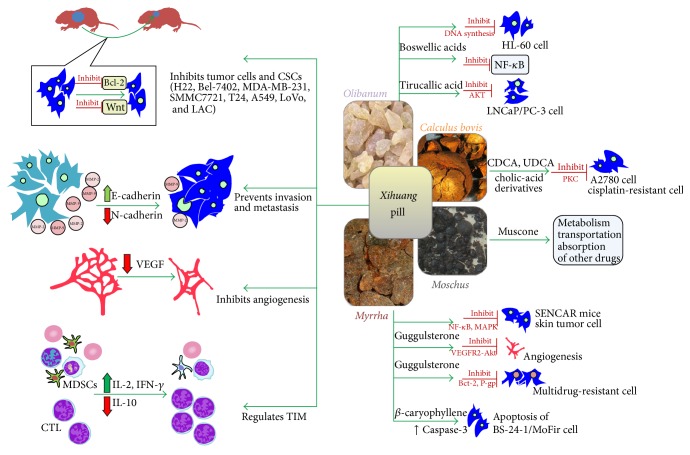
Effects and molecular mechanisms of* Xihuang *pill (XH) and its phytochemicals on tumor treatment. XH could prevent the progression and metastasis of tumors by inhibiting the growth, invasion, and angiogenesis of tumor cells and cancer stem cells. XH can also enhance immunity and reverse the immune suppressive microenvironment (myeloid-derived suppressor cells). Some of the compounds found in XH can inhibit various tumor cells by acting on multiple targets and regulating the metabolism, transportation, or absorption of one another.

*Calculus bovis* has been used in TCM for thousands years to treat high fever, convulsion, inflammation, and tumor.* Calculus bovis* contains bilirubin, bile acids, amino acids, and other compounds. For animal ethics reasons,* Calculus bovis *is identified by its components and artificially synthesized for medical use [96]. Research showed that chenodeoxycholic acid and ursodeoxycholic acid, two bile acids, had significant cytotoxic activity in ovarian cancer cells via induction of apoptosis and reduction of PKC activity [97]. Some new cholic-acid derivatives were synthesized and displayed a distinct cytotoxicity to tumor cell lines [98] (Figure 1).

## 6. Translation and Development of XH

### 6.1. Active Extraction from XH

Phytochemicals have been shown to have effects on tumors [99]. Chemotherapeutic drugs, such as paclitaxel, are extracted and developed from natural compounds [100]. The antitumor effects of monomeric chemicals extracted from XH [84, 92] and their derivatives might be more effective [98]. With further in-depth study and synthetic modifications, we may discover new drugs for tumor treatment.

### 6.2. Research and Development (R&D) Based on Postmetabolic Products of XH

Serum pharmacology is a common method of* in vitro* studies on TCM compound formulas [61], but the chemicals in TCM in serum are unstable and interfere with other factors. Therefore, serum pharmacology is not entirely accepted in TCM basic science.

Extracts or single compounds from TCM contain identifiable compounds, but the compounds have not been metabolized and may have different biological function compared with those in serum [101]. For example, ginsenoside Rb1 has a minimal role in tumor prevention, but ginsenoside 20(S)-protopanaxadiol-aglycone, a metabolite of Rb1, significantly inhibited castration-resistant prostate cancer progression [102]. This might explain why effective clinical TCM compounds can fail in some* in vitro* experiments.

Chinese patent medicines can occasionally attain satisfactory clinical effects on tumors [103] and are becoming increasingly accepted by patients [104]. Further R&D into serum or gastrointestinal postmetabolic products is required, because these compounds could be responsible for the real actions of a TCM. For example, acetyl-11-keto-*β*-boswellic acid (AKBA), one of the most active compounds of olibanum, has numerous metabolites* in vivo* [105] that may affect different targets and cooperate or antagonize one another. Therefore, XH could be more effective if certain active metabolites were selected and others excluded.

### 6.3. High-Level Clinical Evidence for XH Is Necessary

TCM has a recorded history of over 2,000 years that may be used to guide modern treatments for disease and identify neglected but potentially useful treatment strategies [106]. However this process is often based on ancient TCM theories of tradition and history that fail to take into account evidence-based medicine. An increasing number of clinical trials investigating a variety of TCM interventions have been registered in international trial registries, and the design of registered TCM trials has improved by using techniques such as sample size estimation, blinding, and placebos [107]. XH has been shown to have effects on tumors in RCTs and small clinical observations (Table 1). However, more standardized studies should be registered and carried out.

## 7. Conclusion

TCM is based on a set of theories and regards Zheng (syndrome) as the core of a disease [108]. XH is effective for certain syndromes according to TCM and has been shown to have a significant effect on tumors. As a multicompound medicine, XH has multiple targets in tumor treatment and it is needed to farther study how these compounds and their metabolites work together and whether they have synergistic effects with each other. When combined with the conventional medicine XH could be very effective, and XH deserves additional attention in the antitumor research field.

## Figures and Tables

**Table 1:** Clinical applications of XH on tumor therapies.

Diseases	Research method	Combined with/single use	Antitumor effect	Symptoms remission	Ref.
Non-Hodgkin lymphoma	RCT (60 cases)^*∗*^	CHOP regimen	Enhance OS for 3 years	Relieve cancer-related symptoms Enhance KPS	[6]

Breast cancer	(60 cases) RCT (84 cases) (40 cases) (120 cases)	CEF regimen TEC regimen Letrozole Zoledronic acid	Enhance OS for 2 years Enhance RR Enhance FPS Treat bone metastasis	Relieve cancer-related symptoms Relieve endocrinotherapy-induced side-effects Relieve CIBP Enhance KPS Regulate immunity	[14–17]

Primary liver cancer	RCT (80 cases) Clinical observation (23 cases) Clinical observation (28 cases)	TAC (cisplatin) Single use Single use	Enhance RR Enhance OS for 1 year and 2 years	Enhance KPS Relieve cancer-related symptoms	[18–20]

Gastric cancer	RCT (80 cases) Clinical observation (48 cases)^*∗∗*^ Case report (2 cases)^*∗∗*^	DCF regimen Single use Single use	Enhance RR	Relive chemotherapy-induced side-effects Regulate immunity	[21]

Esophageal cancer	RCT (18 cases)	Platinum and fluorouracil based regimens	Could not enhance the effects of chemotherapies	Improve the live quality Relieve cancer and chemotherapy-induced symptoms	[22]

Brain glioma	Case report (1 case)	TCM decoctions	Prolong the survival time to 3 years without undergoing resection Improve the live quality		[23]

Oral mucositis	RCT (60 cases)	Chinese patent medicine	Treat radiation-induced oral mucositis		[24]

Phlebitis	Case report (1 case)	Single use	Treat chemotherapy-induced phlebitis		[25]

^*∗*^The cases number includes the total quantity in the randomized controlled trial (RCT).

^*∗∗*^Unpublished observations.
